# Anti-Inflammatory Activity of Crude Venom Isolated from Parasitoid Wasp, *Bracon hebetor* Say

**DOI:** 10.1155/2017/6978194

**Published:** 2017-10-29

**Authors:** Evelyn Saba, Tahir Shafeeq, Muhammad Irfan, Yuan Yee Lee, Hyuk-Woo Kwon, Myung Gi Seo, Sang-Joon Park, Kyeong-Yeoll Lee, Man Hee Rhee

**Affiliations:** ^1^Laboratory of Veterinary Physiology and Cell Signaling, College of Veterinary Medicine, Kyungpook National University, Daegu, Republic of Korea; ^2^Division of Applied Biosciences, College of Agriculture and Life Sciences, Kyungpook National University, Daegu, Republic of Korea; ^3^Laboratory of Veterinary Histology, College of Veterinary Medicine, Kyungpook National University, Daegu, Republic of Korea

## Abstract

Pest control in the agricultural fields, a major concern globally, is currently achieved through chemical or biological methods. Chemical methods, which leave toxic residue in the produce, are less preferred than biological methods. Venoms injected by stings of various wasps that kill the pest is considered as the examples of the biological method. Although several studies have investigated the biological control of pests through these venoms, very few studies have reported the effects of these venoms on mammalian cells. *Bracon hebetor*, an ectoparasitoid of the order Hymenoptera, is having a paramount importance in parasitizing various lepidopterous larvae including *Plodia interpunctella* also called as Indianmeal moth (IMM). Since it is biologically controlled by *B. hebetor* venom, therefore in our study, herein for the first time, we report the anti-inflammatory activities of the venom from *B. hebetor* (BHV). We developed a septic shock mice model for *in vivo* anti-inflammatory studies and RAW 264.7 cells for *in vitro* studies. Our results clearly demonstrate that BHV can dose dependently abrogate the nitric oxide (NO) production and suppress the levels of proinflammatory mediators and cytokines without posing any cytotoxicity via the nuclear factor kappa B (NF-*κ*B) and mitogen-activated protein kinase (MAPK) pathways.

## 1. Introduction

Parasitoids constitute a powerful weapon for the biological control of lepidopteran insects owing to their venomous and paralyzing stings. One of such parasitoids* Bracon hebetor* Say (HB), occupies a prime place for its parasitic relationship with *Plodia interpunctella* or the Indianmeal moth (IMM). IMM is a widely distributed destructive pest of agricultural produce, which attacks on the final processed product causing huge economic losses to the manufacturers. While many studies in the past have reported various chemical and alternate chemical-based methods to counteract this pest population, the toxic residue left behind by these methods cannot be ignored*. B. hebetor* biologically controls IMM by paralyzing it and causing its death. The chemical nature of BHV which is injected into the host has already been studied in detail. While any kind of venom usually consists of a cocktail of proteins that disrupt the homeostasis of the host, but some venoms such as bee venom are also considered to be a panacea for human ailments [1]. The host-parasite relationship of *P. interpunctella* and *B. hebetor* has been studied extensively in the past, yet no study until now has highlighted the venom's effects in any other context [2–4]. The current study on the BHV was therefore conceptualized based on the already established effects of honey bee venom on mammalian ailments including inflammation and cancer and also its application in human cosmetic industry [5–7].

An invasion of a foreign substance in the body is counteracted by the natural defensive mechanism called “inflammation.” Inflammation is a phenomenon that orchestrates various chemicals in the cells to counteract with the invading material. While inflammation is going on inside the body, it manifests itself externally via signs like swelling, edema, redness, pain, and skin reddening [8]. However, inside the cell, there is production of nitric oxide (NO) that is released under the action of nitric oxide synthase (NOS) enzyme when L-arginine is converted to L-citrulline. Subsequently, a cascade of proinflammatory mediators and cytokines is released that serves to flare up inflammatory process in regard to clear up the foreign antigen. This includes cyclooxygenase-2 (COX-2), interleukins, that is, IL-1*β* and IL-6, and tissue necrosis factor- *α* (TNF-*α*) that attract other inflammatory chemokines as well as to help up for clearance. All these proinflammatory mediators and cytokines lead to activation of classical inflammatory pathway, that is, NF-*κ*B and MAPK pathways [9, 10]. Activation of these pathways regulates a systematic inflammatory response in order to neutralize the invading particle. While inflammation is a natural defense mechanism of the body towards foreign invasion, uncontrolled and generalized inflammatory responses have devastating consequences for the body. Therefore, timely control of inflammation either by the body itself or by an exogenous agent is required.

For this purpose in our study, we tried to unravel the anti-inflammatory activities of BHV on RAW 264.7 cells and in mice model of septic shock. Our results have demonstrated that BHV dose dependently suppressed the nitric oxide (NO) production without any cytotoxicity with suppression in the expression levels of proinflammatory cytokines and mediators both transcriptionally and translationally. Furthermore, it suppresses these effects through the classical NF-*κ*B pathway and mitogen-activated protein kinase (MAPK).

## 2. Methods

### 2.1. Chemicals and Reagents

Dulbecco's Modified Eagle's medium (DMEM) (Daegu, Korea), fetal bovine serum (FBS) (Welgene Co., Daejeon, Korea), streptomycin and penicillin (Lonza, MD, USA), TRIzol® reagent (Invitrogen, Carlsbad, CA, USA), oligo dT (Bioneer Co., Daejeon, Korea), and primers for iNOS, COX-2, TNF-*α*, IL-6, and IL-1*β* were purchased from Bioneer. Lipopolysaccharide (LPS) (*Escherichia coli* 055:B5) and 3-(4,5-dimethylthiazol-2-yl)-2,5-diphenyltetrazoliumbromide (MTT) were procured from Sigma-Aldrich (St. Louis, MO, USA). Specific antibodies against the phospho- and/or total form of IRAK1, TAK1, IKK *α*/*β*, I*κ*B, NF-*κ*B p65, iNOS, COX-2, *β*-actin, and anti-rabbit horse peroxidase-linked secondary antibody were obtained from Cell Signaling Technology (Danvers, MA, USA). All other reagents and chemicals used were obtained from Sigma-Aldrich.

### 2.2. Procurement of Crude Venom Extract from *B. hebetor* Female Wasps

The procurement, purification, and characterization of the venom were according to Quistad et al. and Moreau and Asgari [11, 12]. Briefly, *B. hebetor* were specifically reared and selected from laboratory culture and immobilized on ice for few minutes. The venom glands from each female wasp were then dissected under a microscope and transferred to double-distilled water (DDW). At a single time, batches of 10 glands were dissected and transferred to 50 *μ*L of DDW, homogenized manually, and centrifuged at 12,000 rpm for 5 min at 4°C. The supernatant containing the crude venom was then stored at −20°C for future experimental use. Venom proteins for the experimental use were quantified according to Bradford assay.

### 2.3. Ethical Statement

All animal care and experimental procedures were carried out in strict accordance with internationally accepted guidelines on the use of laboratory animals (IACUC) and the protocols were approved by the Animal Care Committee of the College of Veterinary Medicine, Kyungpook National University, Daegu, South Korea (permit number: 2017–36). For acute LPS treatment, we monitored mice for 96 hours for total and every 12 hours for mortality. After 96 hr, all the remaining mice from the survival study were euthanized with excessive CO_2_ inhalation. For chronic LPS study, we monitored mice for 24 hr total and 12 hours generally for any mortality or other pathology. Our all experimental time was estimated to be around 2 weeks including acclimatization.

### 2.4. Animal Experiments

Male ICR mice 6–8 weeks old (26–29 g) were purchased from Charles River, Orient Biotechnology, Gyeonggi-do, South Korea. The mice were housed in a specific pathogen-free barrier facility at 21 ± 2°C with a relative humidity of 60 ± 10% under a 12 hr light and dark cycle. Feed and water were provided ad libitum. The mice were divided into 3 groups with each group (*n* = 10) for survival study with 30 mg/kg LPS and (*n* = 6) for clinical studies with LPS 20 mg/kg. For survival and clinical studies, venom was given i.p. every day for 5 days prior to LPS treatment. The grouping was group 1: basal (vehicle treatment), group 2: LPS (30 mg/kg or 20 mg/kg), group 3: (venom 30 *μ*g/kg + LPS).

### 2.5. Cell Culture

Murine macrophage cell line RAW 264.7, originating from American Type Culture Collection (ATCC, TIB-71), was cultured in Dulbecco's Modified Eagle Medium (DMEM) supplemented with 5% fetal bovine serum (FBS) (Welgene Co., Daejeon) and 100 IU/mL penicillin and 100 *μ*g/mL streptomycin sulfate (Lonza, MD, USA) with the incubating conditions of humidified 5% CO_2_ incubator at 37°C. The cell line was directly obtained from the company.

### 2.6. Nitric Oxide (NO) Measurement

Griess reaction was the principle for measuring nitric oxide (NO) production. In short, RAW 264.7 cells were seeded in 96-well plates at the density of 2 × 10^4^ cells/well and incubated with or without LPS (0.1 *μ*g/mL) in absence or presence of BHV (0.1–0.4 *μ*g/mL) for 18 hr. The cell culture supernatants (100 *μ*L) were then mixed with Griess reagent (0.2% naphthylethylenediamine dihydrochloride and 2% sulphanilamide in 5% phosphoric acid) in double-distilled water (DDW) at equal volumes and incubated for 5 min at RT (25°C). The absorbance in each well was then analyzed at 540 nm in a microplate reader (VersaMax, Molecular Devices, LLC, CA, USA).

### 2.7. Cell Viability (MTT) Assay

Cytotoxic effects of BHV were studied by measuring the cell viability via MTT assay. Briefly, MTT reagent, that is, 3-(4,5-dimethylthiazol-2-yl)-2,5-diphenyltetrazolium bromide was added to culture medium in 96-well plates at a final concentration of 0.1 mg/mL. After 4 hr of incubation at 37°C in 5% CO_2_, the violet-colored crystals were homogenized in dimethyl sulfoxide (DMSO) 100 *μ*L/well and absorbance was measured at 560 nm.

### 2.8. RNA Extraction and qRT-PCR

For transcriptional studies, RAW 264.7 cells were pretreated with BHV at given concentrations for 30 min and then stimulated with LPS (0.1 *μ*g/mL) for 18 hr. TRIzol reagent was harvested for total RNA extraction both from cells and tissues (Invitrogen, Carlsbad, CA, USA) following the manufacturer's instructions. Subsequent steps were carried out as previously described [13]. Quantitative sequence of primers used for PCR is given in Table 1.

### 2.9. Western Blot Analysis

For direct evidence of signaling pathways followed by BHV, RAW264.7 cells were treated with BHV (0.1–0.4 *μ*g/mL) in the presence and absence of LPS (0.1 *μ*g/mL). NE-PER® Nuclear and Cytoplasmic Extraction Reagents (Thermo Fisher Scientific Co., LTD, Korea) was used for separation of nuclear and cytosolic fractions from cells and tissues. Preceding steps were according to Saba et al. [14].

### 2.10. TNF*α*, NO, and MDA Assay

The plasma was harvested from septic shock mice after euthanasia and then was subjected to different commercially available kits, that is, TNF-*α* ELISA (R&D systems), NO, and MDA (Abcam), according to the manufacturer's instructions.

### 2.11. Hematoxylin and Eosin (H&E) Staining

The lungs and testis tissue after euthanasia to mice were collected in 10% neutral buffered formalin, and then tissues were processed for basic H&E staining according to established protocols [15].

### 2.12. Transient Transfection and Luciferase Assay

Luciferase activity in HEK 293T cells (ATCC, CRL 1573) was measured via the calcium-phosphate method. Briefly, cells were seeded in 60 mm at a density of 5 × 10^5^ cells/plate and incubated for 24 hr and then transfected with TK Renilla (pRL-TK) and NF-*κ*B firefly luciferase (pNF-*κ*BLuc) constructs. The transfected medium was replaced with the normal FBS supplemented media after 6 hr and then again incubated for 18 hr. The next day, cells were seeded in 24-well plates at the density of 5 × 10^4^ cells/well and incubated again overnight. They were then pretreated with BHV (0.1–0.4 *μ*g/mL) for 30 min before PMA stimulation for 6 hr. The following day, cells were quantified for luciferase activity using Promega's Dual-Glo luciferase assay kit (Promega Corporation, WI, USA) according to the manufacturer's instructions. GloMax Luminometer (Promega) was used for measuring luciferase activity. Luciferase activity was normalized to TK Renilla activity. Transcriptional activity of AP1 was also studied similarly.

### 2.13. Statistical Analysis

Data was statistically analyzed using one-way analysis of variance (ANOVA) and Dunnett's test. Values are presented as mean ± SEM. ^∗∗^*p* < 0.05 and ^∗∗∗^*p* < 0.001 were considered statistically significant.

## 3. Results

### 3.1. LPS-Induced Inflammation Was Suppressed by *B. hebetor* Venom

**Figure 1:**
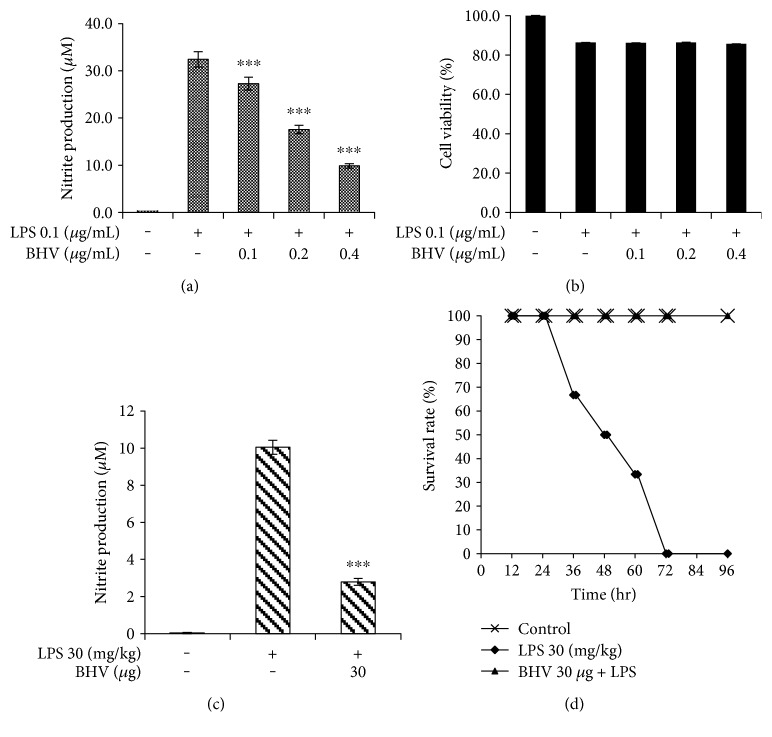
*B. hebetor* venom suppressed LPS-mediated NO release without any cytotoxicity. RAW 264.7 cells were seeded in 96-well plates for 18 hr and then preincubated with BHV (0.1 *μ*g/mL–0.4 *μ*g/mL) for 30 min and then stimulated with LPS (0.1 *μ*g/mL) for 18 hr. The cell supernatant was transferred to a 96-well plate and reacted with equal amounts of Griess reagent, and then NO production was measured at 540 nm (a). Effects of BHV on cell viability were measured by MTT assay and absorbance was measured at 560 nm (b). NO production was also observed in mice chronically treated with LPS (c). For survival rate, mice were given LPS injection with a lethal dose of 30 *μ*g/kg and monitored for 96 hours (d). Values in bar graph are mean ± SEM of at least 3 independent experiments. ^∗∗∗^*p* < 0.001 compared to LPS only.

The component of the gram-negative bacteria's cell wall that is a universal inflammatory inducer is the lipopolysaccharides (LPS) [16]. Therefore, they are used in both *in vitro* and *in vivo* studies as inflammation-inducing agents. Hence, in our study, we investigated whether BHV affects LPS-induced NO production in RAW 264.7 cells and septic shock mice model. As can be seen in Figure 1(a), BHV potently suppressed the LPS-induced inflammation without showing any cytotoxic effect for concentration range used in this study (Figure 1(b)). Moreover, in the plasma of mice given chronic dose of LPS, NO was suppressed (Figure 1(c)) and the venom-treated group showed excellent survival rate of 100% in mice given with a lethal dose of LPS (Figure 1(d)).

### 3.2. *B. hebetor* Venom Inhibited the Expression of Proinflammatory Mediators

**Figure 2:**
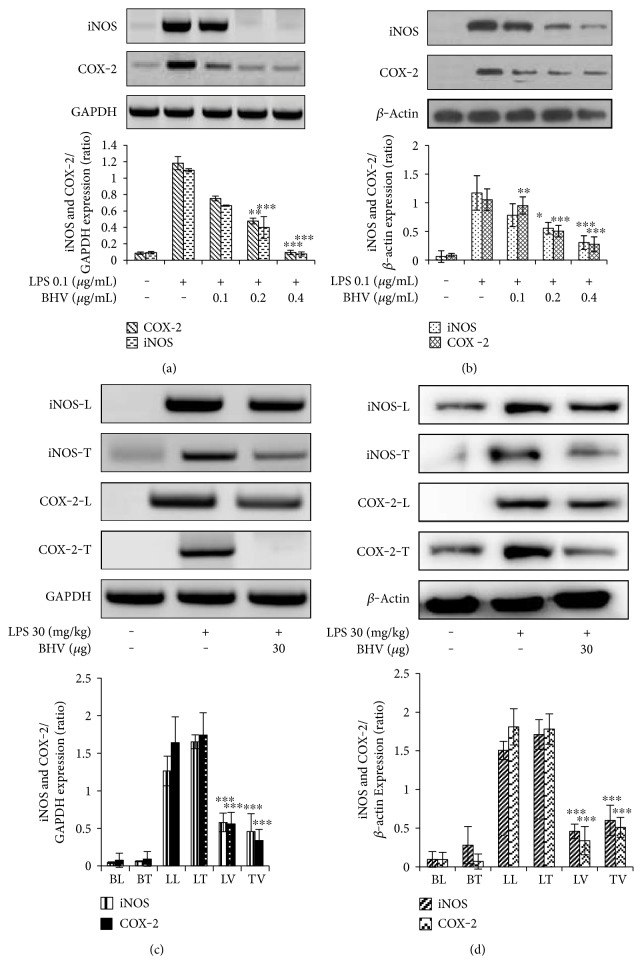
Inhibitory effects of *B. hebetor* venom on the expression of iNOS and COX-2. For mRNA and protein expressions, RAW 264.7 cells were seeded in 6-well plates and treated with indicated concentrations of BHV (0.1 *μ*g/mL–0.4 *μ*g/mL) for 30 min and then stimulated with LPS (0.1 *μ*g/mL) for 18 hr. Total RNA from cell, lung, and testis tissues and total protein was extracted using their respective kit protocols. PCR product was analyzed on agarose gels with GAPDH as the internal control. Protein quantitation was done by Pro-Measure (iNtRON biotechnology, Daejeon, Korea), and then proteins were run on SDS-PAGE and analyzed by ECL chemiluminisence with *β*-actin as internal control. PCR and protein expression in RAW 264.7 cells (a-b). PCR and protein expression in lungs and testis tissue (c-d). Western blot and PCR images are representative of 3 independent experiments. Bar values of ^∗^*p* < 0.01, ^∗∗^*p* < 0.005, and ^∗∗∗^*p* < 0.001 were considered statistically significant. BL = basal lung; BT = basal testis; LL = LPS lung; LT = LPS testis; LV = lung venom; TV = testis venom.

Proinflammatory mediators are the components in the cell that flares up or mediate inflammation. The two most common and rapidly expressing proinflammatory mediators are iNOS and COX-2. We therefore analyzed BHV's effects on proinflammatory mediators' expression. As evidenced by Figures 2(a), 2(b), 2(c), and 2(d), BHV inhibited both the transcriptional and translational expression levels of these components as clearly shown by PCR and immunoblot analysis in both cells and lung and testis tissue of mice which were given chronic LPS dose.

### 3.3. Diminution of Proinflammatory Cytokines *B. hebetor* Venom

**Figure 3:**
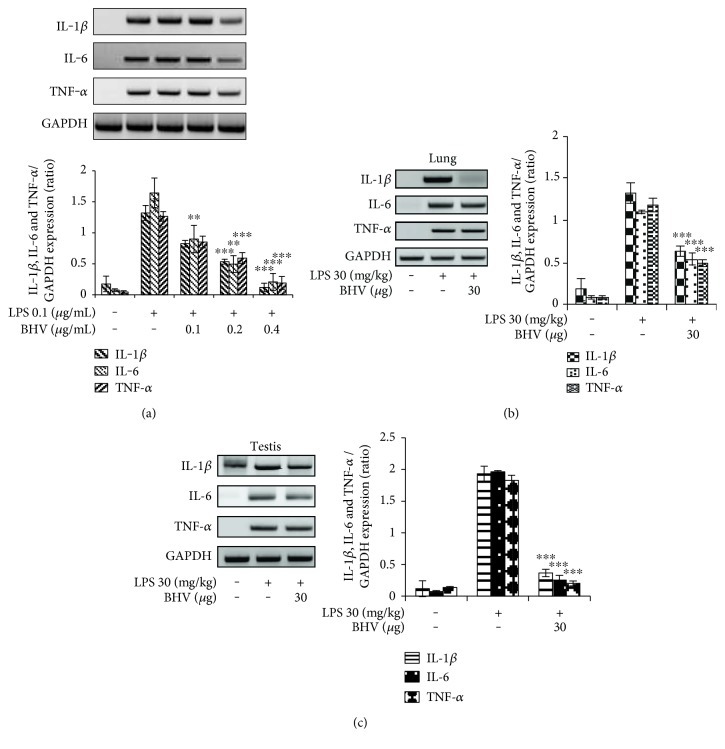
*B. hebetor* venom diminished mRNA expressions of IL-1*β*, IL-6, and TNF-*α* under LPS stimulation. For mRNA expression, RAW 264.7 cells were pretreated with BHV (0.1 *μ*g/mL–0.4 *μ*g/mL) for 30 min and then stimulated with LPS (0.1 *μ*g/mL) for 18 hr. Total RNA from cell, lung, and testis tissue was isolated by TRIzol RNA extraction reagent, and mRNA expression of IL-1*β*, IL-6, and TNF-*α* was determined by RT-PCR. GAPDH was used as the housekeeping gene. Images are representative of 3 independent experiments. Cytokines expression in cells (a). Cytokines expression in lungs (b). Cytokines expression in testis (c). Values in bar graph are mean ± SEM of 4 independent experiments. ^∗∗∗^*p* < 0.001 and ^∗∗^*p* < 0.005 compared to LPS only.

**Figure 4:**
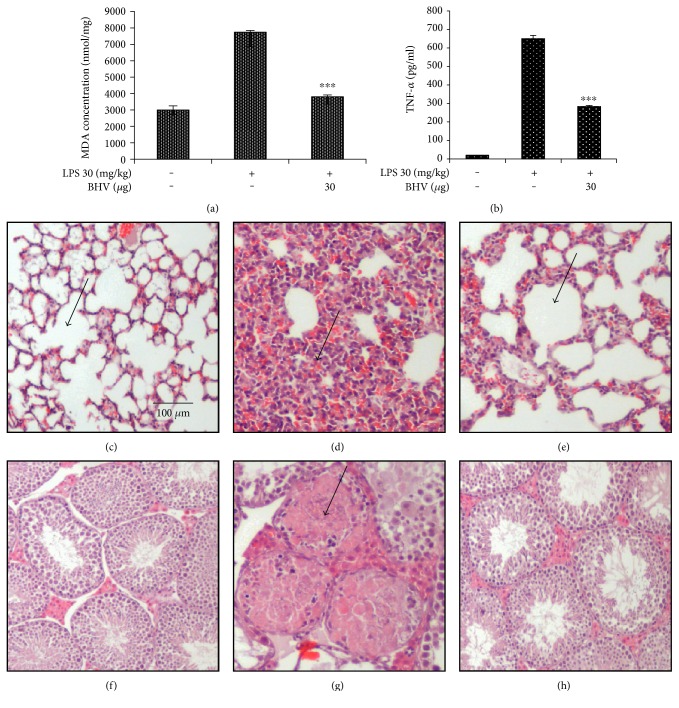
Reduction of serum TNF-*α* and MDA levels by BHV. For the chronic study of septic shock in mice by LPS, mice were treated with venom for 5 days and then were given the LPS injection. 3 days later, they were euthanized and blood and tissues were collected for TNF-*α* and MDA levels by commercially available kits. TNF-*α* levels (a) and MDA levels (b). Values in bar graphs are mean ± SEM of three independent experiments. ^∗∗∗^*p* < 0.001 is considered significant compared to LPS group only. For hematoxylin and eosin staining, lung and testis tissues were stored in 10% neutral buffered formalin (NBF) and then stained according to standardized protocol for (H&E) staining. Lungs (c–e) and testis (f–h). Scale bar 100 *μ*m.

Proinflammatory cytokines are the chemicals that are released in response to inflammation induced as a result of a foreign invader. They serve to increase the magnitude of inflammation and if uncontrolled can lead to severe septic shock. Figures 3(a), 3(b), and 3(c) clearly shows that BHV diminished the mRNA expression levels of IL-1*β*, IL-6, and TNF-*α* in a sharp dose-dependent manner in both cells and tissues. Furthermore, we analyzed the TNF-*α* and MDA levels in the serum of septic shock mice model and found that they both were strongly inhibited in the BHV-treated group (Figures 4(a) and 4(b)). We also geared to check the histology of lungs and testis tissue which is more prone to LPS shock.  Figure 4(c) shows the normal histology of the lungs with clear alveolar spaces and no infiltration of inflammatory cells. However, Figure 4(d) shows the septic shock prone mice with almost no alveolar space that is filled with inflammatory cells. But this histology is reverted back to almost normal by venom treatment as shown in Figure 4(e). In case of the testis tissue, Figure 4(f) shows the normal histology of the testis with proper alignment of cells and subsequent formation of spermatozoa in the seminiferous tubules. In Figure 4(g), however, the seminiferous tubules are degenerated because of LPS shock. But with venom treatment, they appear to regain their normal morphology (Figure 4(h)).

### 3.4. NF-*κ*B and MAPK Pathways Are the Major Players in *B. hebetor* Venom Anti-Inflammatory Activity

**Figure 5:**
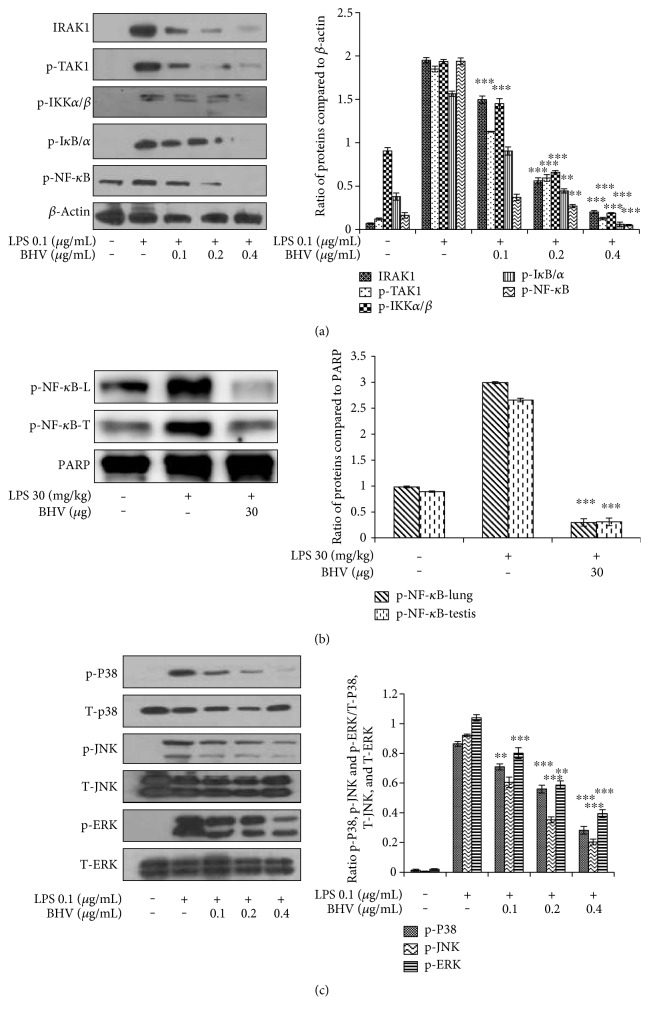
NF-*κ*B and MAPK pathways are followed by *B. hebetor* venom. RAW 264.7 cells were pretreated with BHV at the indicated concentrations (0.1 *μ*g/mL–0.4 *μ*g/mL) and then stimulated with or without LPS (0.1 *μ*g/mL) for 18 hr. Nuclear and cytosolic proteins from cells and tissues were extracted using NE-PER extraction kit. Proteins were run on SDS-PAGE and treated with primary antibodies overnight followed by incubation with secondary antibodies for 2 hr. Expression of proteins in cells (a). Expression of proteins in lung and testis tissue (b). MAPK pathway proteins expression (c). The membranes were analyzed by ECL chemiluminescence system. Data are mean ± SD (*n* = 3); ^∗∗^*p* < 0.005 and ^∗∗∗^*p* < 0.001 compared with LPS only. *β*-actin was taken as internal control.

NF-*κ*B pathway is the universal cascade of transcriptional factors that is followed by any substance for eliciting its anti-inflammatory properties. It is activated when toll-like receptor 4 activation (TLR-4) binds with LPS thus causing the downstream activation of events [17].  Figure 5(a) depicts how BHV has diminished the phosphorylation of interleukin receptor-associated kinase 1 (IRAK1) and transforming growth factor beta-activated kinase 1 (P-TAK-1) which are the foremost initiating factors for NF-*κ*B pathway. Then preceding downwards, BHV has suppressed the phosphorylation of IKK*α*/*β* that degraded I*κ*B/*α*. I*κ*B/*α* then sets NF-*κ*B free from its adjacent subunits and translocates to the nucleus where its phosphorylation had been strongly inhibited by BHV. Moreover, in the lung and testis tissues, NF-*κ*B is also shown to be strongly inhibited with venom treatment (Figure 5(b)). MAPK pathway is also a very commonly activated inflammatory pathway in times of stress to cells. This consists of extracellular signal regulating kinase (ERK), c-Jun N-terminal kinases (JNK), and p38. These factors contribute to the elevation in the expression of activator protein 1 (AP-1) in the nucleus that regulates the production of various genes related to inflammation and immunity [10]. Our results have shown that BHV had dose dependently inhibited the phosphorylation levels of these all three basic components in the MAPK pathway indicating that not only NF-*κ*B but also MAPK is affected by these venom proteins (Figure 5(c)).

### 3.5. Abrogation of Transcriptional Activity of NF-*κ*B and AP-1 by *B. hebetor* Venom

**Figure 6:**
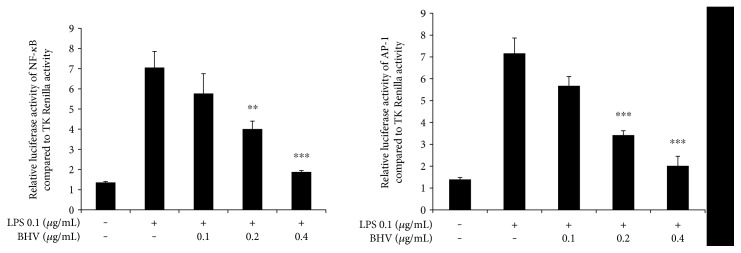
Effects of *B. hebetor* venom on NF-*κ*B and AP-1 transcriptional activities. HEK 293T cells were cultured in 24-well plates and after 18 hr of incubation were transfected with NF-*κ*B, AP-1, and TK Renilla plasmids using the calcium-phosphate method. 48 hr after transfection, cells were pretreated with indicated concentrations of BHV (0.1 μg/mL–0.4 μg/mL) and then stimulated with PMA (0.1 μM) for 6 hr. Luciferase activity was later measured using Dual-Glo luciferase assay system. NF-*κ*B and AP-1 activity was normalized to TK Renilla activity and concentrations were compared to PMA Renilla luciferase activity. Bar graph is mean ± SEM of triplicates. ^∗∗∗^*p* < 0.001 and ^∗∗^*p* < 0.005 were considered statistically significant.

To conclusively establish the direct evidence for *B. hebetor* venom signal transduction via NF-*κ*B and MAPK pathway, we transfected their respective plasmids in the HEK 293T cells and found the expression binding levels via luciferase assay. As shown in Figure 6, BHV had dose dependently diminished the expression levels of both NF-*κ*B and AP-1 (which is the activating transcriptional factor of MAPK in the nucleus) strengthening the possibility that indeed the BHV venom mediated its potent anti-inflammatory effects via the NF-*κ*B and MAPK pathways.

## 4. Discussion

The mechanism through which parasitoids serve in the biological control for decreasing agricultural pests is via paralyzing their host for long or short terms, which interrupts with their developmental or immune processes [12, 18–22]. Previously, many studies had always been limited to the host-parasite relationship studies especially in the context of agricultural pests. This could be attributed to the fact that venoms either from arthropods or reptiles are considered to be dangerously toxic and are severe inflammation-inducing agents. Bee venom however in this regard can be considered as a novel extract that has been studied extensively for its outstanding anti-inflammatory, anti-tumorous, and immune boosting properties [23–26]. Keeping this in mind and also the fact that hymenopteran venoms consist of numerous antimicrobial peptides that can be easily injected in the mammalian subjects to serve as antibiotics, we geared to check the anti-inflammatory activities of BHV [27–29]. Some studies in the past had also investigated the antinociceptive effects of these venom proteins that cause the occlusion of ion channels thus reducing the pain threshold [30, 31].

Different kinds of proteins are present in the composition of any kind of venom. Protein profiles for *B. hebetor* venom showed that it consists of three major amino acids with their molecular weight ranging around 73 KDa. Previously, we have investigated the biological control of IMM via *B. hebetor* on the transcriptional level of genes that are involved in the immunity, metabolism, and development of this pest. However, in regard to the venom itself, we conducted our study to check its anti-inflammatory effects on mammalian cells at very low dosages [12].

Our results indicate that BHV has inhibited the NO production in a dose-dependent manner without any cytotoxicity for our dosage range. NO is a natural defensive gaseous chemical that is produced in response to the foreign invasion in the body or cells. During the combat between foreign particles and cells, their levels are always upregulated which show the first sign of inflammatory reaction in the body. Although its production is good for the rapid neutralization of foreign invader, yet if uncontrolled, it starts destroying the neighboring healthy cells [32].

Proinflammatory mediators are those genes that are responsible for the downstream signaling for activation of inflammatory pathways. Among them, the two most commonly encountered mediators are iNOS and COX-2. In our results, BHV has potently inhibited the expression levels of both of these mediators both at the transcriptional and translational levels indicating that BHV has the potency to inhibit the expression of agents causing inflammation.

After proinflammatory mediators, proinflammatory cytokines come into the play. These are the chemicals responsible for causing the local or systemic inflammation depending upon their timely control and levels of a foreign invader. The first three proinflammatory cytokines are mustered out, the IL-1*β*, IL-6, and TNF-*α* [33]. The release of these proinflammatory cytokines serves to flare-up the inflammatory process that is being suppressed by BHV in our results. Timely control through an endogenous or exogenous agent is critical to control these chemicals as it can lead to systemic infection in no time [34].

We have discussed the release of NO, proinflammatory mediators, and cytokines previously, but there are some pathways that cause the production of these agents from an extracellular to intracellular environment. Nuclear factor kappa B pathway (NF-*κ*B) is one of the classical inflammatory pathways that is activated whenever there is some foreign invasion in the cell. This pathway comprises a series of transcriptional components from the cytoplasm and nucleus that is responsible for the induction of inflammation. NF-*κ*B signaling initiates the stimulation of IRAK1 that phosphorylates TAK1 which acts upon IKK*α*/*β* that is present at dormancy in the cytoplasm. Activated phosphorylation of IKK*α*/*β* stimulates I*κ*B*α* subunit of NF-*κ*B setting it free to translocate into the nucleus [35–37]. In our results, BHV has dose dependently suppressed the phosphorylation expression of every component related to classical NF-*κ*B and MAPK pathways.

## 5. Conclusion

In conclusion, BHV has inhibited the phosphorylation of all factors involved in both of the above-stated pathways both *in vitro* and *in vivo*. Therefore, BHV can be reckoned as a strong anti-inflammatory agent in future for advanced molecular studies.

## Figures and Tables

**Table 1:** Sequence of primers used for RT-PCR for *B. hebetor* venom gene expression.

Gene	Primer	Oligonucleotide sequence (5′-3′)
GAPDH	F	5′CAATGAATACGGCTACAGCAAC3′
	R	5′AGGGAGATGCTCAGTGTTGG3′

iNOS	F	5′CCCTTCCGAAGTTTCTGGCAGCAGC3′
	R	5′GGCTGTCAGAGCCTCGTGGCTTTGG3′

COX-2	F	5′-TCTCAGCACCCACCCGCTCA-3′
	R	5′-GCCCCGTAGACCCTGCTCGA-3′

IL-1*β*	F	5′CAGGGTGGGTGTGCCGTCTTTC3′
	R	5′TGCTTCCAAACCTTTGACCTGGGC3′

TNF-*α*	F	5′TTGACCTCAGCGCTGAGTTG3′
	R	5′CCTGTAGCCCACGTCGTAGC3′

IL-6	F	5′-GTACTCCAGAAGACCAGAGG-3′
	R	5′-TGCTGGTGACAACCACGGCC-3′

## Abbreviations

BHV: *B. hebetor* venom
IMM: Indianmeal moth
LPS: Lipopolysaccharides
NO: Nitric oxide
iNOS: Inducible NO synthase
TNF-*α*: Tumor necrotic factor alpha
COX-2: Cyclooxygenase-2
NF-*κ*B: Nuclear factor kappa B
MAPK: Mitogen-activated protein kinases
TLR: Toll-like receptors
IL: Interleukin
ERK: Extracellular signal-regulated kinases
JNK: c-Jun N-terminal kinases
IKK: Inhibitor of kappa B kinase
I*κ*B/*α*: Inhibitor kappa B-alpha.
